# Association of the Specimen and Tumor Bed Margin Status with Local Recurrence and Survival in Open Partial Laryngectomy

**DOI:** 10.3390/jcm13092491

**Published:** 2024-04-24

**Authors:** Rogério Aparecido Dedivitis, Leandro Luongo de Matos, Mario Augusto Ferrari de Castro, Luiz Paulo Kowalski

**Affiliations:** 1 School of Medicine, University of São Paulo, São Paulo 05508-220, Brazil; dedivitis.hns@uol.com.br (R.A.D.); l.matos@fm.usp.br (L.L.d.M.); lp_kowalski@uol.com.br (L.P.K.); 2 School of Medicine, Metropolitan University of Santos, Santos 11045-002, Brazil

**Keywords:** margins of excision, laryngectomy, laryngeal cancer, carcinoma, squamous cell, frozen sections, recurrence, survival rate

## Abstract

**Background/Objectives**: Positive margins are associated with locoregional recurrence in early laryngeal cancer. The aim of this study was to evaluate the impacts of specimen-driven (ex vivo) positive margins on patients with early-stage laryngeal cancer whose tumor bed (defect-driven) margins had been negative. **Methods**: A retrospective study was performed on 60 consecutive T1b/T2 glottic cancer patients who underwent open frontolateral laryngectomy. The intraoperative margins were obtained from the tumor bed. Their recurrence and disease-free survival were evaluated. In all cases, negative margins were obtained from the surgical bed. The impact of positive margins from the specimen was evaluated in a paraffin study. **Results**: Among 10 patients with positive margins in the specimen, six experienced local relapse, and among 50 patients with negative margins in the specimen, three developed recurrence. The 5-year disease-free survival rates were 37.5% and 93.9%, respectively (*p* < 0.001; log-rank). Even with negative margins in the surgical bed, patients with positive margins in the specimen at the final histopathological examination had a 3.5-fold higher chance of developing local recurrence than those with negative margins (HR = 13.993; 95% CI: 3.479–56.281; *p* < 0.001; univariate Cox regression). **Conclusions**: Specimen-driven positive margins represent a significant risk factor for local recurrence, even under negative margins at the tumor bed.

## 1. Introduction

The success of the surgical excision of head-and-neck squamous cell carcinomas relies on completely resecting the tumor with negative margins. The histopathological examination of formalin-fixed, paraffin-embedded specimens (i.e., permanent samples) is the gold standard for assessing the completeness of resection. The presence of malignant cells on the excision surface is related to tumor transection. Thus, viable tumor cells are assumed to have been left behind, indicating positive margins [1].

There are many reasons for positive margins; however, they remain one of the prognostic factors in head-and-neck cancer that are under the direct view of the surgical team. During surgery, the surgeon depends on the macroscopic characteristics of the tumor (both visual and tactile) to assess the extent of the lesion in order to achieve a wide remotion [1].

Frozen-section analysis is considered to be the diagnostic gold standard for the intraoperative pathological evaluation of surgical margins to ensure the complete resection of the tumor. Positive margins are strongly associated with the risk of local recurrence and mortality, influencing decisions regarding postoperative adjuvant therapy [2].

There are two means of margin assessment: sampling of the tumor bed (defect-driven) and sampling of the excised specimen ex vivo (specimen-driven). These approaches vary among different surgeons and departments, particularly in cases of laryngeal squamous cell carcinoma. No consensus has been achieved to date. Some studies favor the specimen-driven margin method, since it has been associated with superior local control and oncological outcomes [2]. However, a 2005 AHNS study demonstrated that only 14% of surgeons who answered the questionnaire preferred a specimen-driven evaluation, whereas the remaining 76% preferred to obtain tissue from the surgical bed [3].

If frozen sections show initially positive margins, however, on additional resection negative margins are confirmed and most surgeons consider that tumor to have negative margins. For tongue cancer, some studies indicated that such an assumption may not be accurate. Even so, the use of frozen sections to evaluate tumor margins is generally accepted. Out of 476 completed surveys received, 99% of answers indicated the use of frozen sections to verify tumor margins. According to the different anatomic sites, the percentage of surgeons who used frozen sections varied a lot. In fact, more than 90% used frozen sections in the evaluation of oral cavity and pharynx tumors. It contrasted with around 20% to 50% in other sites of the head and neck.

Tumors originating in the oropharynx, hypopharynx, and larynx also yield resection specimens that are three-dimensionally complex. The unnecessary resection of wide margins is not advisable, since it can impact vocal outcomes and the related quality of life, especially in early-stage laryngeal cancer [2].

Negative frozen sections obtained intraoperatively with positive margins in the paraffin-embedded pathology report can increase morbidity and mortality for these patients. These “false negative” frozen sections can be an indication for further treatment modalities, such as re-resection and (chemo)radiotherapy. As a result, additional treatment costs can be incurred, resulting in further financial burdens on the healthcare system [4].

There is a lack of studies focusing on margin status in open partial vertical laryngectomy. For open approaches, the majority of papers regarding head-and-neck cancer focus on oral cancer. The papers regarding laryngeal cancer often refer to transoral laser microsurgery, and some papers have evaluated the role of margins in supracricoid horizontal laryngectomy.

The findings of a cross-sectional study suggest that intraoperative frozen-section histopathology is accurate in comparison with the definitive pathological characteristics of the same specimen in oral cancer. However, it is not so reliable in assessing the permanent margin status of the tumor sample. Despite its high specificity, the sensitivity is quite low. This can be attributed to the limitations of the intraoperative margin analysis of the tumor bed in accurately considering the three-dimensional relationship of tumor margins with the periphery of the specimen, along with the generally low rate of positive definitive tumor margins. Although intraoperative frozen-section histopathology is widely used in approaches to oral tumors, there are limitations to this modality in evaluating the final status of the obtained operation margins [5]. A significant debate in the literature remains concerning the optimal site for obtaining frozen sections from head-and-neck tumors. It is, therefore, debatable whether it is appropriate to take samples from the resected oral cavity tumor or the surgical site bed. At this tumor site, there is increasing evidence that a specimen-driven evaluation reliably leads to a more precise definition of margins and is associated with higher rates of local control and overall survival [6,7]. Several studies have recommended a specimen-oriented model of intraoperative frozen-section analysis, reporting decreased false negative rates and improved patient outcomes in oral cancer [4]. However, there is a lack of similar studies for laryngeal squamous cell carcinoma.

The status of margins significantly affects local control in laryngeal squamous cell carcinoma treated with transoral laser microsurgery. In a meta-analysis of nine studies with 3130 patients, positive margins had an aHR of 1.3, with a CI ranging from 0.56 to 2.97. Thus, the impact of positive margins on overall survival could not be defined [8]. In another meta-analysis of 16 studies with 2808 T1-T2N0M0 patients who underwent laser cordectomy, the recurrence rate in patients who presented positive margins was higher (*p* = 0.003) in comparison to those with negative margins. A good collaboration between the pathologist and surgeon was recommended in order to diminish the rates of false positive margins [9]. In fact, among patients treated with transoral laser microsurgery, positive margins can be identified in up to 50% of them. Moreover, a high rate of false positive findings—up to 80%—has been reported. Close and positive margins are associated with higher recurrence rates in comparison to negative margins [10].

Supracricoid partial laryngectomy is a horizontal approach that reduces the indications for total laryngectomy in selected cases of advanced laryngeal cancer. In comparison with vertical partial techniques, its local control and long-term organ preservation rates are higher. Supracricoid laryngectomy presents a higher local control rate in stage I laryngeal cancers in comparison to the other treatment options. Open vertical laryngectomy has good oncological outcomes for T2 or T3 glottic cancers; however, the oncological outcomes of horizontal techniques are superior for selected glottic stage II cancer. Supracricoid laryngectomy is not the method of choice for non-salvage approaches to early laryngeal cancer due to the large resection of healthy anatomical structures; however, there are some studies about the impact of its resection margins. A retrospective study of 253 patients who underwent supracricoid horizontal laryngectomy found that a positive resection margin was the sole predictor of local control under univariate and multivariate analysis [11]. In a retrospective study of 63 patients who underwent supracricoid laryngectomy, positive excision margins were significantly associated with recurrence in both univariate and multivariate analyses. Positive margins also showed a significant association with overall survival under univariate analysis [12].

The aim of this study was to evaluate the impacts of specimen-driven positive margins in patients with early-stage squamous cell carcinoma who underwent open vertical laryngectomies and whose final tumor bed margins were reported as negative.

## 2. Materials and Methods

A retrospective cohort of consecutive patients with T1b/T2 glottic squamous cell carcinoma with extension to/through the anterior commissure, treated by open vertical frontolateral laryngectomy as the primary surgery was evaluated.

This study received approval from the Institutional Review Board of the institution under the number 426.662 (CAAE 13497813.3.0000.0068), and patient informed consent was waived due to the retrospective nature of the study.

Patients met the inclusion criteria if they received histopathological confirmation of squamous cell carcinoma, clinically staged as glottic T1b/T2, and were treated with open surgical resection (frontolateral laryngectomy) of the primary tumor without neck dissection. Patients were excluded if there was inadequate clinical or pathological information for analysis, or if they had received previous oncological treatment(s) on the neck.

Data were extracted from the patients’ medical records. Collected data included demographic characteristics (age, sex, tobacco and alcohol consumption), clinicopathological features (clinical staging and margin status), and outcomes (local recurrence and calculated disease-free survival). All tumors were staged using the eighth edition of the American Joint Committee on Cancer [13].

The general practice at our institution is to take intraoperative frozen-section margins from the residual tumor bed rather than from the specimen. Both circumferential margins and deep margins (obtained from the deep muscle boundaries) were evaluated. Thus, the intraoperative margins were obtained from the tumor bed, and the final histopathological paraffin study samples were obtained from the surgical specimen and by revision of the frozen sections’ fragments. In cases of intraoperative positive margins from the tumor bed, they were re-excised until definitive negative margins were obtained. For close margins (less than 1 mm), re-resections were performed on a case-by-case basis, depending on the surgeon’s judgment of morbidity and the feasibility of re-resection. Thus, all patients had a report of negative margins from a pathological evaluation of frozen sections at the end of the resection, regardless of the need for re-excision.

The definitive postoperative report of the paraffin-embedded surgical specimen was reviewed. For the paraffin-embedded specimens’ margin results, a binary classification system was developed, with histopathological reports interpreted as either negative (negative, atypia, or dysplasia) or positive (carcinoma in situ, suspicious, or positive for invasive carcinoma). Definitive margins of the specimen were considered negative if they were at least 2 mm clear of disease according to the pathologist’s evaluation.

The definitive status of the specimen margins was studied according to recurrence and disease-free survival, considering a follow-up of at least three years. Disease-free survival was calculated between the date of the surgery and the date of local recurrence (or the last follow-up date for those who did not present this outcome).

Surgical technique: A curved transverse neck incision was performed and the skin flaps were elevated. A protective tracheotomy was created without communication with the surgical bed. The external perichondrium of the thyroid cartilage was lifted back by 2 cm. Incisions were made in the thyrohyoid membrane and in the cricohyoid membrane, close to the limits of the midline of the thyroid cartilage. The larynx was entered, and two parallel incisions were made lateral to the midline of the thyroid cartilage. The specimen was resected under direct view. This included the vocal fold with the tumor, the anterior commissure attached to the cartilage around its midline, and part of the contralateral vocal fold. Samples were obtained from the tumor bed for the examination of frozen sections. After obtaining clear margins, the epiglottis petiole and the preserved vocal folds were sutured in the new anterior commissure. The perichondrial layer and the bipedicle sternohyoid muscle were used to reconstruct the inner larynx. They were positioned in the larynx inside the resected ipsilateral vocal fold, and the closure was carried out using planes [14,15].

Statistical analysis: The values obtained from the quantitative variables were organized and described as the mean and standard deviation (SD), as well as the minimum and maximum values. Absolute and relative frequencies were used for qualitative data. Kaplan–Meier curves were used in the survival analyses, and the log-rank test was applied to compare the curves. A univariate Cox proportional regression model was used to estimate the hazard ratio (HR) and 95% confidence interval (95% CI) values. A *p*-value of 5% or less (*p* ≤ 0.05) was adopted as the threshold for statistical significance. All statistical tests were performed using SPSS 29.0 software (IBM^®^ Inc; Endicott, NY, USA).

## 3. Results

This study included 60 patients, 55 of them males (91.7%), with a mean age of 62.5 years (SD = 9.1; minimum = 40; maximum = 85). There were 55 (91.7%) chronic smokers and nine (15%) heavy drinkers. Regarding the clinical stage, 54 (90%) patients were T1bN0M0, whereas six (10%) patients were T2N0M0.

All patients underwent open frontolateral laryngectomy and had final negative margins upon the examination of frozen sections from the tumor bed, with further re-excision if necessary. Thus, four patients (6.7%) underwent re-excision using the same procedure due to positive margins upon frozen-section examination. On the other hand, the rate of positive margins in the surgical specimens upon the final histopathological paraffin-embedded examination was 16.7% (10 cases).

**Figure 1:**
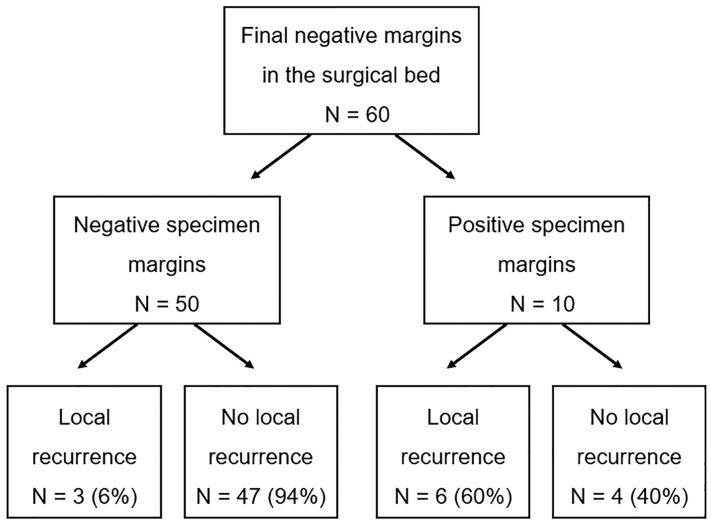
Distribution of the patients and the recurrence findings based on the status of the margins.

**Figure 2:**
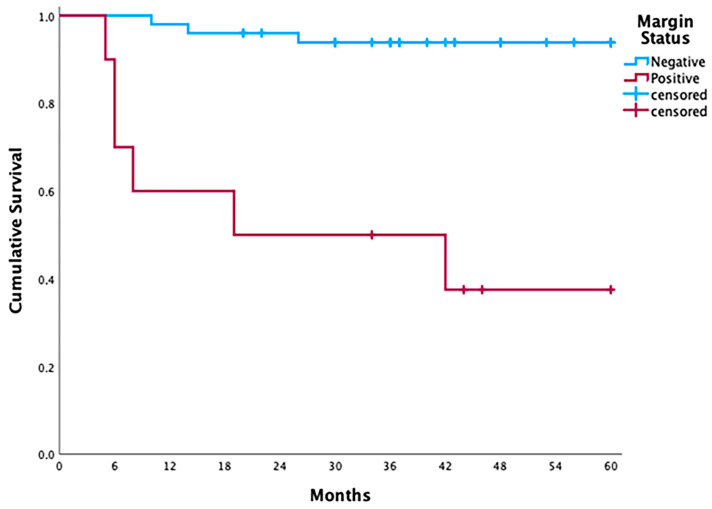
Kaplan–Meier curves illustrating a lower cumulative local recurrence-free survival rate in patients with positive margins at the final histopathological examination compared to those with negative margins (37.5% vs. 93.9%, respectively, *p* < 0.001—Log-Rank test).

Of the 10 patients who presented positive margins upon the final paraffin-embedded histopathological examination of the surgical specimens, six (60%) developed local relapse during the follow-up; of these six patients with local relapse, two underwent a salvage total laryngectomy, and all patients received radiotherapy. The cumulative disease-free survival was 37.5% for this group of patients, as well as a median survival of 19 months. Only three patients (6%) with negative margins in the surgical specimen developed recurrence, reaching a 5-year cumulative disease-free survival of 93.9% (*p* < 0.001; log-rank test) (Figure 1 and Figure 2).

Finally, the patients with positive margins in the surgical specimen upon the final histopathological examination had a higher risk of developing local recurrence than those with negative margins (HR = 13.993; 95% CI: 3.479–56.281; *p* < 0.001; univariate Cox regression model).

## 4. Discussion

We evaluated a retrospective cohort of patients with T1b/T2 glottic squamous cell carcinoma treated by open vertical frontolateral laryngectomy as the primary treatment. Definitive negative margins were obtained in all tumor beds after the excision. Those with positive margins in the surgical paraffin-embedded specimen presented a higher risk of developing local recurrence than those with negative margins in the specimen.

There is a lack of studies on margin status after an open partial laryngectomy. On the other hand, this technique has a well-established role in the treatment of early glottic tumors with transoral laser microsurgery, with a high likelihood of local control and good functional outcomes in cases of negative margins. This technique is an effective and safe approach for patients with early glottic tumors. Resection with a narrow oncological margin used to be the goal to preserve as much tissue as possible. The resection margin is around 1–2 mm according to the clinical aspect of the tumor. In fact, in recent decades, there has been a decline in the use of open partial laryngectomy as a first-line treatment for laryngeal cancer [16]. The surgical margins after the resection of early glottic carcinoma are often very close or equivocal since there is a trend toward sparing as much tissue as possible for functional reasons. As a result, the margins are difficult to analyze [17]. A study evaluated the prognostic value of positive intraoperative frozen sections, analyzing 75 patients. Initially, positive frozen-section margins were significant predictors of recurrence, even after the re-excision of the cordectomy field to obtain negative margins [18]. Final histological analysis is often less favorable than the extemporaneous analysis for discovering positive margins [19]. During endoscopic surgery, the surgeon must deal with the difficulties of the histological examination due to small-fragment analysis and tissue shrinkage. The intraoperative findings should be considered in the decision to propose further treatment or monitoring. In the event of a macroscopically satisfactory surgery, the surgeon is more likely to follow the patient than to propose additional treatment if the margins are close or equivocal. Some practical problems for performing frozen sections were related. This method is not available in some centers. Moreover, extemporaneous small fragment analysis can be unreliable [17]. High rates of indeterminate margins have been reported after transoral laser microsurgery, ranging from 17.2 to 33% [20]. Tumor bed biopsies after the initial resection are considered an alternative to intraoperative frozen sections since they can predict the local recurrence independent of the margin status of the specimen. However, false negative results can occur due to sampling errors [21]. The large quantity of positive or equivocal margins does not reflect clinically a corresponding rate of recurrences or therapeutic failures following endoscopic approaches. In our study, the findings were quite different in a series of patients who were submitted to an open approach.

In oral cancer, there is some trend toward a specimen-oriented model of intraoperative frozen-section analysis; however, there is a lack of published data on open vertical laryngeal approaches to date.

After the resection of the main specimen, there are two different approaches to obtain intraoperative margins: to take samples from the tumor bed, targeting areas that are assumed to be at risk (defect-driven); or sending the entire resection specimen to the pathologist for the examination of all faces of the specimen (specimen-driven) [6]. In the former situation, the specimens must be oriented anatomically, which requires an evaluation of the three-dimensional aspects and landmarks. This approach allows the pathologist to assess the full surface of the tumor–margin interface; however, it is more labor-intensive [2]. Although a consensus has not yet been reached, the literature has been in favor of the specimen-driven margin approach, as it has been linked to improved local control [3,22,23,24]. Another benefit of specimen-driven margins is the ability to measure the distance from the tumor to the margin. Conversely, defect-oriented margins can only be reported as positive or negative [25].

In oral cavity cancer, the prognosis improves if at least 5-mm margins are achieved. However, 50% to 80% of negative margins are achieved among those patients. Sampling of tissue from the surgical bed after excision presents potential caveats. First, the assessment of the distance between the surgical margin and tumor cells is not feasible, but the involvement or not of cancer can be verified. The identification of tumor cells is hard since the specimens miss the core of the tumor. Moreover, electrocautery artifacts and random sampling errors can represent some limitations in identifying cancer [22]. In a literature review, 10 studies evaluated the intraoperative assessment of resection margins in soft tissue in oral cancer. Defect-driven results presented an average accuracy, sensitivity, and specificity of 90.9%, 47.6%, and 84.4%. On the other hand, specimen-driven results were 91.5%, 68.4%, and 96.7%, respectively. The majority of the specimen-driven studies performed a gross examination of the mucosal and deep margins, followed by an analysis of locations considered suspicious for inadequate margins [23].

The surgeon and pathologist must agree on the main specimen orientation and areas concerning a close or positive margin in oral cancer. There are some possible advantages of the tumor bed-driven approach. Some surgeons prefer to be in charge of the anatomically correct designation of margins since they know where they came from. Some surgeons prefer to obtain the frozen sample before the tumor is thoroughly out. Pathology reports could be further controlled. The concept of the mucosal and deep margin must be clear for the team, as is the submucosal spread of a tumor to achieve an adequate resection specimen [24].

In oral cancer, the use of tumor-bed-driven frozen sections did not show benefits for the re-resection of an intraoperative positive frozen section. In a retrospective cohort study of 406 patients with oral cancer, local recurrence was identified in 36% of the patients when an invasive carcinoma was present at an intraoperative frozen margin, and in 45% when an invasive carcinoma was found on the permanent specimen margin, compared with 19% and 13%, respectively, for completely negative frozen and permanent margin findings. There was a significant difference in local recurrence rates between patients with negative margins on both intraoperative and permanent specimens (13%) and those with positive intraoperative margins subsequently cleared by additional resection to negative margins (27%), as well as between the first group and those with negative intraoperative but positive permanent specimen margins (34%); however, there was no difference between positive intraoperative margins cleared by additional resection and negative intraoperative but positive permanent specimen margins. Thus, clearing positive frozen margins from the tumor bed does not improve outcomes in oral cancer [26]. By studying early laryngeal cancer in a cohort of patients who underwent open partial vertical laryngectomy, we found similar results: 60% of the patients who presented positive margins at the final histopathological examination and 6% of those with final negative margins developed local recurrence, and the cumulative 5-year disease-free survival rates were 37.5% and 93.9%, respectively.

One limitation of this study is that it is retrospective. Even considering that this series of T1b/T2 glottic cancer was consecutive, some bias can be present, since other prognostic factors could not be evaluated, such as comorbidities. However, one can consider its generalizability, at least to patients receiving this specific surgical approach—open frontolateral partial laryngectomy. Our hypothesis is that the positive margins from the tumor specimen may have a negative prognostic impact, even when margins from the patient after the resection are clear. It is in agreement with some mentioned studies on oral cancer. As a result, despite being less commonly employed than endoscopic resection, such findings possibly could be generalized to other surgical techniques for the treatment of laryngeal cancer.

## 5. Conclusions

Positive margins in specimen-driven postoperative pathological evaluation in the presence of negative intraoperative margins from the tumor bed represent a significant risk factor for local recurrence. This suggests that in the future, samples should be taken from the specimen instead of the tumor bed for intraoperative frozen sections in open partial laryngectomy for the treatment of early laryngeal cancer.

## Data Availability

All data is included in the article.
